# Eosinophilic Esophagitis as a Side Effect of Food Oral Immunotherapy

**DOI:** 10.3390/medicina56110618

**Published:** 2020-11-16

**Authors:** Antonella Cianferoni

**Affiliations:** Perelman School of Medicine, University of Pennsylvania, Medical Director Food Allergy Immunotherapy Program, The Children’s Hospital of Philadelphia, ARC1202B, 3615 Civic Center Boulevard, Philadelphia, PA 19104, USA; Cianferonia@email.chop.edu; Tel.: +1-267-294-1952

**Keywords:** eosinophilic esophagitis, oral immunotherapy, food allergy

## Abstract

Food allergies (FAs) include a spectrum of immune-mediated serious and potentially life-threatening medical conditions with an overall estimated prevalence ranging from 4% to 8% in the U.S. and Europe. Significant progress in food allergen-specific immunotherapy has been accomplished over the past 10 years. The most studied strategy has been oral immunotherapy (OIT), also known as food desensitization, a treatment in which a child is slowly and deliberately given a small amount of the food to ingest (that previously was a food allergy trigger) with the ultimate goal of the child eating that food without a reaction. OIT is now recommended in the European guidelines for the treatment of milk, egg, and peanut allergies and was the first American Food Drug Administration (FDA) approved product for the prevention of severe reaction to peanuts in 4–17 year olds to be released on the market. The side effects associated with OIT treatment trials are mild to moderate, predominantly oropharyngeal, and easily treated. More severe reactions, such as generalized urticaria/angioedema, wheezing/respiratory distress, laryngeal edema, and repetitive emesis, have been reported. However systemic reactions are very rare. Low-dose immunotherapy is associated with significantly fewer side effects. Currently, its most limiting allergic side effect is that approximately 10–15% of subjects treated with OIT experience gastrointestinal symptoms, preventing the continuation of therapy. Eosinophilic esophagitis (EoE) has also been reported as a cause of persistent abdominal symptoms in OIT.

## 1. Introduction

Food allergies (FAs) include a spectrum of immune-mediated serious and potentially life-threatening medical conditions with an overall estimated prevalence that ranges from 4% to 8% in the U.S. and Europe [1,2]. Even more concerning is the fact that FA incidence has increased dramatically in the last 20 years; for example, peanut allergy has increased from 0.8% to 2.5% [2,3]. In the U.S., children treated at our hospital, the Children’s Hospital of Philadelphia (CHOP), have some type of food allergy in 7.2% of cases [4].

FAs are pathological, immunologic, and reproducible reactions to allergens contained in one’s diet [5]. Like all atopic inflammation, FAs are largely due to a Th2-specific inflammatory process and are based on the importance of resulting Immunoglobulin E class (IgE) production in their pathogenesis [6]. FAs can be further classified into (a) IgE-mediated; (b) non-IgE-mediated when the specific IgE to foods is not important and the cell compartment of the immune system is responsible of the food allergy; (c) mixed IgE-/cell-mediated when both IgE and immune cells are involved in the reaction [7,8,9,10].

The most well understood and common FA is the IgE-mediated FA to common allergenic foods such as milk, eggs, peanuts, tree nuts, seafood, soy, and wheat. They are triggered by allergen-specific antibodies of the IgE class that bind IgE high-affinity receptors (FcƐRI) expressed on basophils and mast cells. When an allergen crosslinks two receptors, it activates the cells to release histamine and other mediators, which induce vasodilation and/or smooth muscle constriction with consequent hives, angioedema, low blood pressure bronchospasm, diarrhea, and vomiting [11]. Specific IgE can be detected by in vitro or in vivo diagnostic methods, and together with history and food challenges, confirm an FA diagnosis [5]. Treatment for IgE-mediated FA (IgE-FA) is the avoidance of the allergens, the availability of rescue medications such as self-injectable epinephrine, and, lately, immunotherapy.

Non-IgE-mediated food allergies (non-IgE-FA) have been increasing worldwide in parallel to IgE-mediated ones. The most common non-IgE-FA is eosinophilic esophagitis (EoE), but other known disorders include non-EoE eosinophilic gastrointestinal disorders (non-EoE-EGID), food protein-induced enterocolitis (FPIES), and food protein-induced allergic proctocolitis (FPIAP).

Non-IgE-FAs represent immunologic reactions to food and occur in the absence of food-specific pathogenetic IgE antibodies in the skin or serum; therefore, they can have several different pathogenetic mechanisms [12]. Although Th2 cells and other innate immunity cells (i.e., epithelial cells, mast cells, basophils, invariant natural killer T cells, and innate lymphocytes) have been shown to play an important role in EoE, the pathogenesis of FPIES and FPIAP is still not understood [13,14].

## 2. Food Allergy Oral Immunotherapy (OIT)

An IgE-FA occurs immediately after ingestion and is associated with severe and sometimes life-threatening allergic reactions (e.g., anaphylaxis) [3,5]. In high-risk cohorts, an accidental exposure results in approximately one reaction per subject per year, consequently resulting in hundreds of Emergency Department (ED) visits, hospitalizations, and approximately 20 deaths per year in the U.S. [15]. In children and young adults, the majority of anaphylactic reactions treated in the emergency room are triggered by foods [15,16,17,18,19,20]. Indeed, 70% of anaphylaxis cases that have been seen in the CHOP Emergency Department in the last year were due to food [19]. It is not currently possible to predict which individuals are at risk of life-threatening reactions based on their history of level/type of IgE [21]. Until recently, the treatment of FAs was solely based on avoidance of the allergy triggers and maintaining access at all times to self-injectable epinephrine to promptly treat any anaphylaxis due to accidental exposure to the food allergens [3,5]. Developing effective treatment strategies outside of the dietary avoidance of allergens and the availability of self-injectable epinephrine has been a high priority for families, advocacy groups, funding agencies, and research teams. Significant progress has been accomplished in food allergen-specific immunotherapy over the past 10 years [1]. The most studied strategy has been oral immunotherapy (OIT), also known as food desensitization, a treatment in which a child is slowly and deliberately given a small amount of food to ingest (that previously was a food allergy trigger) with the ultimate goal of the child eating that food without a reaction [22,23].

OIT is now recommended in the European guidelines for the treatment of milk, egg, and peanut allergies and was the first FDA-approved product for the prevention of severe reaction to peanuts in 4–17 year olds to be released on the market [24,25]. Sublingual immunotherapy and epicutaneous immunotherapy are still under investigation [1].

In the last decade, approximately 900 studies, including many randomized multicenter, placebo-controlled, clinical trials, have provided valuable, in terms of their efficacy and safety, data for the evaluation of OIT as an active treatment [26,27,28,29,30,31,32,33,34,35,36,37]. OIT has been shown to be effective in 60–80% of research trials [1,38].

Herein, we highlight some examples. In a trial of peanut OIT, 28 children (aged 1–16 years) were randomized to receive peanut OIT (4000 mg) versus placebo OIT [36]. Peanut OIT was associated with increased peanut consumption compared to the placebo after 12 months (5000 vs. 280 mg, *p* < 0.001), and with decreased skin prick test (SPT) size and TH2 cytokine levels, as well as increased peanut-specific IgG4 levels and regulatory T cell (Treg) numbers [36]. Similar findings were noted during a milk OIT trial in 20 children with a milk allergy (aged 6–21 years), randomized to receive milk OIT (500 mg) versus a placebo [34]. After six months, the change in the reaction threshold was 5100 versus 0 mg in the OIT *(p <* 0.002) and placebo *(p* < 0.16) groups, respectively.

Other randomized controlled trials for milk OIT in children have shown similar clinical findings [33,39,40,41]. In a study from the Consortium of Food Allergy Research (CoFAR), 55 children with an egg allergy (aged 5–18 years) were randomized to receive egg versus placebo OIT [29]. In 40 subjects receiving egg OIT, 55% passed a 5 g Oral Food Challenge (OFC) at 10 months versus 0% of the placebo-treated subjects (dose consumed, 5000 vs. 50 mg; *p* < 0.001); meanwhile, 75% of the subjects receiving egg OIT passed a 10 g OFC at 22 months.

Generally, the side effects associated with OIT treatment trials are mild to moderate, predominantly oropharyngeal, and easily treated. More severe reactions, such as generalized urticaria/angioedema, wheezing/respiratory distress, laryngeal edema, and repetitive emesis, have been reported. However, systemic reactions are very rare [25,42]. Low-dose immunotherapy is associated with significantly fewer side effects [1,25,26]. Currently, its most limiting allergic side effect is that approximately 10–15% of subjects treated with OIT experience gastrointestinal symptoms, preventing the continuation of therapy [22,23,25,42]. EoE has also been reported as a cause of persistent abdominal symptoms in OIT.

## 3. Eosinophilic Esophagitis

EoE is a clinical pathologic disease characterized by eosinophilia limited to the esophagus and symptoms of esophageal dysfunction [43,44]. The typical symptoms of EoE are dysphagia and food impaction due to esophageal fibrosis; however, these specific symptoms are frequent in adults but not in children, who tend to present with aspecific symptoms related to esophageal inflammation, such as failure to thrive, feeding difficulties, gagging, vomiting, and food refusal [13].

When the clinical picture is suggestive of EoE, diagnosis is based on a biopsy obtained via esophagogastroduodenoscopy (EGD) if it shows at least 15 eosinophils per high-power field (eos/hpf) [45,46,47]. All diagnoses and follow-ups in EoE are based on endoscopic findings. Multiple biopsies need to be taken, even from a macroscopically normal esophagus, with four to six being the optimal number to reduce the false negative rate [48,49,50].

Patients with EoE have a high rate of atopic comorbidities (i.e., allergic rhinitis, asthma, an IgE-mediated food allergy, and/or eczema) [51]. FAs appear to play a role in over 90% of children and adults with EoE, as elemental diet is able to induce clinical pathologic remission in all patients [52,53,54,55].

The most common food triggers in EoE are milk and wheat, but eggs, legumes, meats, and soy can also initiate inflammation alone or in combination [13], probably by activating Th2 cells [6]. A dysfunctional epithelium may also contribute to the pathogenesis of EoE by facilitating allergen penetrance and by inducing a local and possibly systemic Th2 response against food and environmental allergens in genetically susceptible individuals [13,56].

In order to ameliorate the symptoms, to increase quality of life, and to prevent irreversible fibrosis, treatment needs to be started as soon as an EoE diagnosis is confirmed. There are three main clinically accepted clinical treatment strategies for EoE: proton pump inhibitors (PPIs), corticosteroid treatment, and dietary elimination. Mechanical dilation is an option in patients with irreversible fibrosis [57].

PPIs at a high dose (1 mg/kg in children and 40 mg of omeprazole or equivalent) once or twice a day are effective in approximately 20–50% of patients [58] and are generally considered the first drug to be used in the management of EoE [59,60]. Those patients responsive to a PPI can be treated indefinitely with said PPI [59]. Therefore, a therapy with dietary exclusion or swallowed steroids will only be started in those patients that do not respond to PPIs [59]

Meanwhile, steroids can lead to clinical and histopathological remission of EoE. Oral steroids are effective in virtually all patients; however, their significant long-term side effects limit their use in brief emergency treatment [61]. Topical corticosteroids such as fluticasone and budesonide have also been shown to control inflammation in up to 95% of patients [62]. Oro-dispersible budesonide tablets have been approved in Europe for use in adults; otherwise, oral viscous budesonide or swallowed fluticasone from a metered dose inhaler are used off label [63,64]. No food or drink is allowed 30 min after the medication is administered.

FAs are now globally recognized as the most well-known trigger of EoE; indeed, various types of elimination diets have been successfully used in EoE management [61,65,66,67,68,69,70,71,72]. The more restrictive the diet, the more rapid the success in a large percentage of patients. Diets based solely on an amino-acid-based formula are effective in over 90% of patients, while single-food elimination diets are successful in 30–60% of patients, with a diet based on the elimination of two to six foods showing intermediate efficacy [61,69,73].

Ultimately, most patients are allergic only to one or two foods, so the diet should be tailored to the specific patient so as to reduce the limitation and to maximize the efficacy. The most restrictive diets, such as elemental and six-food elimination diets, are associated with a significant reduction in quality of life and they are extremely difficult to adhere to in the long term, requiring a very high number of EGDs to normalize the diet [61,65,66,67,68,69,70,71,72,74]. Two-food elimination (i.e., milk and wheat) in a recent prospective study was shown to be preferable to more restrictive approaches [61,65,66,67,68,69,70,71,72].

Despite all of the limitations, empiric diets are still the most effective option in EoE, as allergy testing, such as measurements of specific IgE (in vivo via a skin prick test (SPT), or in vitro via serum analysis) and atopy patch tests, have been shown to be less effective than the empiric method to find the food trigger of EoE [59,75]. This is probably due to the fact that the role of IgE in food-induced EoE is minimal, if any [65], and to the lack of standardization of food atopy patch tests [75].

## 4. OIT Risk of Developing EoE

Atopic comorbidities are very frequent in patients with EoE [61,76,77]. Various studies have shown that up to 80% of EoE patients are atopic (23–27% have IgE-mediated food allergy, 38–60% have asthma, 60–64% have allergic rhinitis, and 18–46% have atopic dermatitis) [77,78,79]. In comparison, in the U.S., 4–6% have IgE-mediated food allergy, 8–10% have asthma, 30–40% have allergic rhinitis, and 3–20% have atopic dermatitis [80,81,82].

In particular, FAs show a strong link to EoE. Children with FAs, especially those with multiple IgE-FAs, develop EoE in over 5% vs. 0.1% of the general population in the U.S. [83]. Interestingly, the risk of developing EoE persists as children outgrow IgE-FAs and reintroduce previously non-tolerated foods into the diet [84].

If food regularly present in the diet can trigger EoE, it is not surprising that OIT and sublingual allergen immunotherapy (SLIT) have been associated with the development of EoE. Subcutaneous immunotherapy (SCIT), to the best of our knowledge, has not been reported to be associated with EoE. Only isolated case reports of SLIT for dust mite and pollen have been published [85,86,87].

OIT for IgE-mediated food allergies is instead recognized as being more frequently associated with the development of EoE, in agreement with the larger role of food allergens as EoE triggers. In a metanalysis, Lucendo et al. found EoE as a complication of OIT in approximately 2.7% of cases [88]. More recently, Petroni and Spergel [89] closely reviewed 110 OIT studies for various foods (e.g., milk, eggs, and peanuts) and found that 5.7% of patients undergoing OIT developed biopsy-proven EoE. If patients develops EoE during OIT, they are recommended to discontinue the therapy, with usual improvement of clinical and histological manifestations (Table 1).

In a Spanish study [90], out of 128 patients undergoing OIT, eight (6.2%) developed endoscopically proven EoE (six in isolation and two with gastric or colic involvement as well). Two of the patients were updosing, while the other six were in maintenance after 15–47 months of OIT. Six patients were receiving milk OIT, one egg OIT, and one egg and milk OIT. All but one patient (who was found to have EoE incidentally due to a scope for celiac disease) had symptoms: abdominal pain and vomiting were the most common symptoms, while three patients developed dysphagia, and one patient with eosinophilic gastroenteritis developed diarrhea. Two patients discontinued OIT after an improvement in symptoms, while the other six opted to continue OIT and to treat EoE. All patients were treated with PPIs and four with topical steroids as well; two patients treated with PPIs and one treated with PPIs and steroids went into remission during OIT, while one patient clinically improved and refused OIT and one patient treated with PPIs and steroids did not enter remission, despite the therapy. The patients with eosinophilic gastroenteritis also received oral steroids.

Endoscopically diagnosed EoE may underestimate the real incidence of EoE in patients undergoing OIT, of whom up to 34% develop gastrointestinal (GI) symptoms. GI symptoms may spontaneously resolve over time or after OIT modifications, or else may persist and lead to the suspension of OIT. The vast majority of patients who develop GI symptoms do not undergo endoscopy, so we do not know if some of these patients also have EoE. For example, in a study conducted in Israel [91], out of 794 patients undergoing OIT for various foods (i.e., 614 for milk, 130 for peanuts, 41 for eggs, and 9 for sesame), 8.2% (65/794) developed recurrent gastrointestinal symptoms independent of the timing of the administration of the dose (milk, 9.0% (55/614); peanut, 6.9% (9/130); egg, 2.4% (1/41)). Of note, none of the nine patients receiving the sesame OIT developed these symptoms. Of the patients who developed GI symptoms, 72.3% had multiple episodes of vomiting and 27.7% had milder symptoms of abdominal pain (27% of the patients during the milk OIT and 33% during the peanut OIT). In general, these symptoms appeared early in the course of OIT: 38.5% within the first month of home treatment and 86% occurring within the first three months. None of the patients complained of dysphagia or had clinical evidence for food impaction, and the cessation of dosing led to an improvement in symptoms in all patients. Three patients evaluated with EGD had biopsy esophageal eosinophilic counts pathognomonic for EoE (30, 170, 65/hpf) (0.4%). Patients were not previously treated with proton pump inhibitors. The peripheral blood was evaluated for eosinophils for all patients in the study, and patients with high eosinophils tended to have a higher level of GI symptoms. Group 1 (371 patients) had Eos in the blood <900 eos/mm^3^ and only 0.5% developed GI symptoms (0 vomiting); Group 2 (213 patients) had 900–1500 eos/mm^3^ and 15% developed GI symptoms (12% vomiting); Group 3 (88 patients) had 1500–2500 eos/mm^3^ and 27% developed GI symptoms (18% vomiting); Group 4 (135 patients) had >2500 eos/mm^3^ and 46% developed GI symptoms (33% vomiting). Monthly increases in OIT dosage were resumed in 45 cases, with the consumption of a once-daily dose at home. More than 90% of the patients who resumed treatment (42 of 45) did not redevelop symptoms. Further investigations are needed to understand the meaning of peripheral blood hypereosinophilia and the possible indications of ongoing EoE. Indeed, peripheral eosinophilia is an aspecific sign of atopy and does not necessarily correlate with EoE diagnosis [92]. Low-dose OIT seems to be correlated with less severe and less frequent GI symptoms and rare EGD-confirmed EoE (0.26%) [25].

Omalizumab has been shown to reduce the risk and frequency of acute reaction to OIT, but does not appear to be able to prevent EoE development, nor is it an effective treatment for EoE. Indeed, in a study with OIT + omalizumab, 15% of patients (2/13) developed EGD-confirmed EoE. Of note, one of the two patients had symptoms prior to initiating OIT, and EoE did not resolve upon discontinuation of OIT, suggesting that EoE predated OIT initiation [93].

As we learn more, we are discovering that many patients who develop EoE during OIT may have had it prior to the initiation of SLIT or OIT. In asymptomatic adults, esophageal eosinophilia and frank EoE is found in up of 15% of patients with a peanut allergy prior to OIT initiation [94]. This confirms the high incidence of EoE found in patients with FAs [79,83,94]. In a follow up study the same authors randomly assigned those 20 patients to groups given peanut OIT (*n* = 15) or placebo (*n* = 5); and serial gastrointestinal biopsies were collected at baseline (*n* = 21, 0 weeks), following dose escalation (*n* = 10, 52 weeks), and during the maintenance phase (*n* = 11, 104 weeks). Biopsies were assessed for eosinophils per high-power field (eos/hpf) OIT induced or increased esophageal eosinophilia (EE) at 52 weeks in most subjects with 57% of active OIT patients meeting the criteria of EoE (≥15 eos/hpf) with no significant changes in esophageal peak eosinophil counts in the placebo group. However in four of six participants (67%), OIT-induced EE and gastrointestinal eosinophilia resolved by the end of the maintenance phase. Gastrointestinal symptoms were not clearly associated with EE or gastrointestinal eosinophilia [95]. Therefore, in order to determine the true incidence of de novo EoE in patients treated with OIT, prospective studies should be conducted where baseline endoscopy is performed in all patients, with serial endoscopic evaluations throughout treatment or in the event of symptoms. At the moment if symptoms are significant and patients meet the criteria of EoE most clinicians will stop OIT, maybe in the future with a better understanding of the EoE vs. transient EE more patients will be able to be treated under close monitoring.

## 5. Conclusions

**Figure 1:**
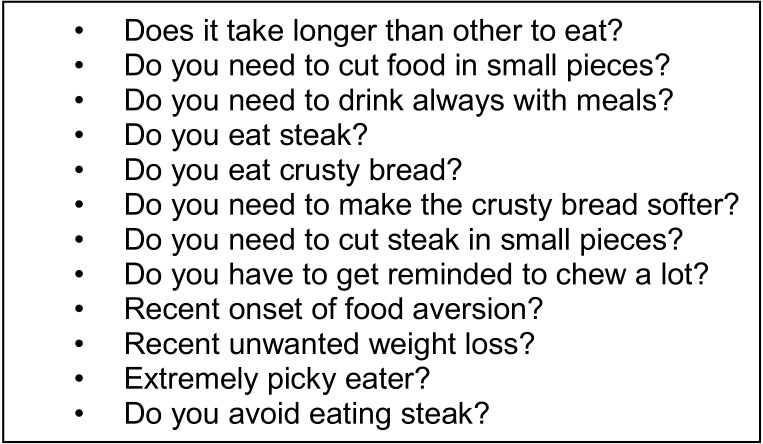
Suggested screening questions for eosinophilic esophagitis (EoE) prior to start oral immunotherapy (OIT).

EoE is a described side effect of food OIT, and children with FAs, especially those with multiple food allergies, are at an increased risk of developing EoE. Therefore, it is important to understand the typical manifestations of EoE in different age groups to recognize the symptoms early. Patients undergoing OIT are at risk of developing various degrees of abdominal pain and to also develop EoE before or after OIT initiation. As we screen patients for asthma or previous life threatening reactions to increase the safety of OIT, it is advisable to screen for the presence of possible symptoms of EoE, another contraindication to start OIT. Children with EoE have typically a 7-year delay in their diagnosis as the early manifestation of EoE are very aspecific, so some patients may have unrecognized disease. Furthermore often another family member is affected and may make symptoms more difficult to recognize as pathologic in the family. It is therefore advisable to ask if children have chronic symptoms of gastroesophageal disfunction (Figure 1) in FA patients especially patients who desire to undergo SLIT or OIT.

Early diagnosis is important because EoE is a chronic illness and needs to be treated as soon as possible in order to prevent fibrosis and long-lasting psychosocial consequences as a result of the inevitable changes in functioning and life style linked to chronic diseases. A collaboration with a GI expert in EoE is essential in children undergoing OIT with persistent belly pain, vomiting, or any sign of dysphagia.

At present, once EoE is diagnosed, OIT is suspended, while EoE prior to treatment is a contraindication to start OIT. This situation will continue until more studies are available for long-term patients with OIT-related EoE.

## Figures and Tables

**Table 1:** EoE in OIT food trials.

Author	Year	Food	Dose	Total pts	EoE	%
Babaie D	2017	Milk	120 cc	18	1	5.6
Goldberg,	2017	Various	Various	794	3	0.4
Echeverría-Zudaire	2015	Milk and or egg	8 g	128	8	6.25
MacGinnitie	2017	Peanut + Xolair	4 g	37	1	2.702703
Palisade	2018	Peanut	300 mg	372	1	0.268817
Burk CM	2017	Peanut + Xolair	4 g	13	2	15.4
MacGinnitie	2017	Peanut + Xolair	4 g	37	1	2.702703
Andorf, S	2018	Multifood- + Xolair	1 g	45	0	0

Pts: patients; EoE: eosinophilic esophagitis; OIT: oral immunotherapy.
